# Contact allergies to topical antibiotic applications 

**DOI:** 10.5414/ALX02253E

**Published:** 2022-02-01

**Authors:** Burkhard Kreft, Johannes Wohlrab

**Affiliations:** University Hospital Halle (Saale), Department of Dermatology and Venereology, Martin Luther University Halle-Wittenberg, Halle (Saale), Germany

**Keywords:** topical antibiotics, antiseptics, contact allergy, epicutaneous patch testing, neomycin, fusidic acid, octenidine, polyhexanide, allergy

## Abstract

Despite limited evidence on clinical efficacy and increasing resistance problems, topical antibiotics are still used in everyday clinical practice. However, topical antiseptic agents such, as octenidine and polyhexanide, often have a broader efficacy spectrum. They also have a broader target tropism because of their non-specific cellular mechanisms of action. Repeated use of topical antibiotics also carries the risk of contact sensitization, which could limit potential subsequent use as systemic antibiotics. Contact allergy is a clinically relevant problem, particularly in patients with barrier-damaged skin, pre-existing dermatosis, or occupational exposure. It can be concluded that with the use of modern antiseptics, topical antibiotic therapy is rarely indicated and should be avoided, not only because of the risk of contact sensitization but also because of the unfavorable and potentially consequential resistance problem.

## Introduction 

Topical antibiotics (AB) are generally used for the treatment of superficial infections of the skin, eye, and ear [1]. Their use in dermatology is also well established in conditions such as acne vulgaris and rosacea [2]. Although local AB are popular in clinical practice, limited evidence on clinical efficacy means that today, prescribing can only be recommended for a few indications [3]. Theoretical advantages of local antibiotic therapy for skin infections are the deposition of a high antimicrobial drug concentration at the site of infection as well as the reduction of the risk of systemic toxicity [3, 4]. In addition, agents that are not available for systemic use can be applied locally [2]. However, the use of topical AB is only useful for superficial (staphylogenic) skin infections, as there is usually insufficient penetration of the active ingredients into deeper skin layers [3]. The use in other diseases, such as rosacea or acne, must be considered in a differentiated manner, since an immunomodulatory rather than an anti-infective effect is intended. In many cases, combined preparations of topical AB and glucocorticosteroid are used unnecessarily for the treatment of inflammatory skin conditions – for example in eczema diseases – without a skin infection even being present [3]. Apart from the limited evidence on clinical efficacy, the usefulness of local antibiotic therapy must be critically questioned because of increasing bacterial resistance [3]. Consistent local antiseptic treatment with active substances, such as octenidine or polyhexanide, often covers a wider spectrum of efficacy. Modern antiseptics also have a broader target tropism because of their non-specific cellular mechanisms of action, and are therefore much less likely to lead to the development of resistance compared with locally applied AB. Long-term or repeated use also increases the risk of contact sensitization (CS) and thus limits the subsequent systemic use of the antibiotic [1]. 

## Contact allergies to topical antibiotics 

The prevalence of contact allergy (CA) is generally quite low but may be underdiagnosed. CA is a clinically relevant problem, especially in high-risk patients [1, 2]. CS resulting from repeated topical antibiotic therapies most commonly develop from uncritical self-treatment, occupational exposure, or are iatrogenic [1]. An overview of antibiotics as a cause of contact allergies can also be found in Table 1. Particular risk factors are the use of topical AB on barrier-damaged skin or in the presence of pre-existing dermatoses. Repeated and occlusive therapy in intertriginous skin areas also increase the risk of contact allergy (CA) [1, 2, 5]. Studies have shown that patients with chronic venous insufficiency, chronic otitis externa, post-operative or post-traumatic wounds, and chronic eczema lesions treated with topical AB are particularly predisposed to the development of allergic contact dermatitis (CD) [5, 6]. AB in topical ophthalmic treatments can lead to periocular eczema as a consequence of CS [7, 8]. CA should be considered if there is no improvement or even a paradoxical worsening of the local findings during therapy with topical AB. In the case of acute allergic CD, edematous, erythematous, and pruritic papules and vesicles appear at the site of contact allergen application [1]. 

CA, as a result of occupational exposure, is often triggered by antibiotics that are usually administered systemically, for example, via improper handling, such as repeated accidental skin contact when preparing antibiotic infusions to be administered systemically (β-lactam antibiotics, especially cephalosporins) without protective gloves [9]. A transfer of the allergen via the hands to another part of the body may result in “dislocated” eczema on forearms or face [5, 9]. Occupationally induced aerogenic CD and generalized exanthema have also been described in the literature [9, 10]. In principle, occupationally induced allergic reactions of the immediate-type with urticaria and anaphylaxis due to antibiotics are also possible and have been described. The risk of sensitization by occupational exposure is not only relevant for healthcare workers, but also for workers in the pharmaceutical industry and in agriculture. Natural and semi-synthetic penicillins are among the more common allergens responsible for allergic CD [10]. In principle, it can be assumed that generalized drug reactions (drug exanthema) in acquired CS are also possible if the corresponding AB or a related substance is taken systemically. De Castro Martinez et al. [12] reported a case of systemic CD to fusidic acid with previous sensitization via the skin. By analogy, it is conceivable that sensitization via systemic administration of an AB also leads to allergic CD on subsequent skin contact [9]. Whether an atopic disposition is increasingly associated with allergic CD remains controversial [13, 14, 15]. The same applies to the risk of CS to topical AB [1]. 

Among topical ABs, aminoglycosides, such as neomycin, gentamycin, and framycetin, appear to have the highest risk of CS [2]; other triggers include bacitracin, chloramphenicol, clindamycin, and erythromycin [1, 16]. Co-sensitization to multiple substances that are structurally unrelated but contained in the same preparation is also possible. This has been repeatedly described for neomycin and bacitracin [16]. 

### Aminoglycoside antibiotics 

Aminoglycoside antibiotics as topical preparations are not only used in dermatology but also in ophthalmology and otolaryngology [6, 17]. A high prevalence of CA to gentamicin is found in patients with chronic venous insufficiency and otitis externa. Another frequently used topical aminoglycoside antibiotic is neomycin, not least because of low therapeutic costs. As a broad-spectrum antibiotic with bactericidal activity against Gram-negative (not effective against *Pseudomonas aeruginosa* and anaerobic bacteria) and Gram-positive bacteria, particularly *Staphylococcus aureus*, neomycin is widely prescribed for the treatment of superficial skin infections [2]. In ophthalmology, it is used in the form of eye drops for bacterial conjunctivitis, and in otolaryngology for the treatment of otitis externa [6]. Unlike in Germany, neomycin (as well as gentamicin) is available in the USA as an over-the-counter preparation in combined steroid formulations (dexamethasone), with antifungal agents (nystatin) as well as together with bacitracin [16, 18, 19]. It is therefore one of the most common contact allergens in North America, with sensitization rates exceeding 8% [6]. An analysis of epicutaneous patch test data in 10 European countries in 2005/2006 showed sensitization rates for neomycin to be between 1.1 and 3.8% [20]. It is also significant as a contact allergen in children (in the USA and in some European countries) [21]. Due to the combined use with bacitracin, there are also many cases of co-sensitization to neomycin and bacitracin. The combination of neomycin and glucocorticosteroid may mask the clinical picture of allergic CD [5]. Because of their structural relationship to each other, aminoglycoside antibiotics are characterized by a high rate of immunologic cross-reactions [16]. Thus, in the case of CS to neomycin, cross-reactions to other aminoglycoside antibiotics, such as amikacin, framycetin, gentamicin, tobramycin, kanamycin, and butirosin, are common [1, 7]. Notably, in epicutaneous patch testing with aminoglycoside antibiotics, the maximum of a positive test reaction is often reached after 7 days, so late readings are very important [6]. In 2019, CA after topical application of paromomycin was reported for the first time by an Italian research group [22]. Streptomycin is used systemically to treat tuberculosis, other mycobacterial infections, and infections caused by enterococci and streptococci. CA to streptomycin is observed in healthcare professionals and pharmaceutical workers [1]. Spectinomycin has a different chemical structure among aminoglycosides and therefore shows minimal cross-reactivity [16].

### Polypeptide antibiotics 

These include the polymyxins and also bacitracin. Because of high toxicity, systemic use is rarely justifiable. Bacitracin is produced by *Bacillus subtilis*, inhibits bacterial cell wall synthesis, is effective against Gram-positive bacteria and is used for the prevention and therapy of superficial skin infections. Because of potential nephrotoxicity, the substance is restricted to topical use [4]. Bacitracin was declared “Contact Allergen of the Year 2003” by the American Contact Dermatitis Society in 2003. Since “late” test reactions are common in epicutaneous patch testing, it can be assumed that CS to bacitracin has been overlooked more frequently in the past [23]. Of note in this context is that immediate-type allergic reactions are also possible. Thus, anaphylaxis has been described in the context of intra-operative bacitracin irrigation [24] or by use in topical preparations [25]. Polymyxin B binds to the cell membranes of bacteria and disrupts their osmotic properties. Its antibiotic activity encompasses Gram-negative bacteria including *Pseudomonas*. For decades, polymyxin B has been used in topical preparations to treat skin, eye, and ear infections, often in combination with other antimicrobial agents. Polymyxin B, bacitracin, and neomycin are sold in combination (“one cream treats all”) as over-the-counter preparations in some countries [26]. Polymyxin B had previously been thought to be a rather weak sensitizer. However, in a retrospective cohort study of 795 patients in Canada (where polymyxin is available only on prescription), in whom epicutaneous patch testing was performed, a prevalence of CS of as much as 2.3% was seen [26]. Moreover, in analogy to bacitracin and neomycin, it could be observed that positive epicutaneous patch test reactions are often not noticeable until day 4, so it is recommended to always perform late readings with topical AB [23, 26]. Although bacitracin and polymyxin B are cyclic polypeptides, they differ in chemical structure in a way that immunologic cross-reactions are not very likely [26]. Virginiamycin is another cyclic polypeptide that is occasionally used in Europe for topical treatment of infections with Gram-positive bacteria. It is still used as a growth promoter in cattle, pigs, and poultry in some countries and thus may cause occupational contact dermatitis in livestock workers [1]. Pristinamycin is a related streptogramin AB. Factor M of virginiamycin is identical to fraction IIA of pristinamycin. Therefore, cross-reactions of the two ABs are common [1]. However, virginiamycin and pristinamycin are hardly used in humans today. 

### Lincosamide antibiotics 

Clindamycin is a semi-synthetic derivative of lincomycin, inhibits bacterial protein synthesis, and is effective against aerobic Gram-positive cocci and some anaerobic and microaerophilic Gram-negative and Gram-positive micro-organisms [1]. Topical applications of clindamycin include the treatment of acne vulgaris and bacterial vaginosis. However, despite frequent long-term use, it is rarely a trigger of allergic contact dermatitis [27]. CA to a 1% alcoholic clindamycin solution for the treatment of facial acne was first described in 1978 [28]. Since acne therapy often involves the use of a variety of other topical preparations, a diagnosis is often delayed. Manifestations of atypical clinical pictures of CA, such as rosacea-like rash [29] or erythema multiforme-like skin lesions [30]**,** have been described. Therefore, in the case of a paradoxical worsening as well as a change of the clinical appearance of acne despite therapy, CA should always be considered [28]. Immunological cross-reactions between clindamycin and lincomycin, which is not approved for human medicine in Germany, are possible. 

### Macrolides 

The macrolide antibiotic erythromycin inhibits bacterial protein synthesis. It is actively effective against most aerobic and anaerobic Gram-positive and some Gram-negative bacteria. Topically, erythromycin is used in the treatment of acne vulgaris, rosacea, and perioral dermatitis as well as superficial skin and eye infections. The occurrence of allergic CD is very rare [2, 31]. 

### Beta-lactam antibiotics 

β-lactam antibiotics inhibit mucopeptide synthesis in the bacterial cell wall. They are rarely used in topical pharmaceuticals. Previously reported cases of CA often relate to healthcare workers and the pharmaceutical industry or pharmaceutical manufacturing. Among semi-synthetic penicillins, ampicillin is an occasional cause of occupational CA [1]. CS to cephalosporins is also more commonly found in the context of occupational exposure. Immediate-type allergic reactions have also been described [11]. Immunologic cross-reactions within a cephalosporin group of the same generation are common. Knowing the sensitizing potential of penicillin, topical use is now widely avoided. In Malaysia, where topical penicillin was formerly available as an over-the-counter agent, it was the most common cause of AB-induced allergic contact dermatitis in 1976 [32]. Cross-reactions between penicillins, semi-synthetic penicillins, and cephalosporins are theoretically possible due to the common β-lactam ring, but are rarely observed in practice because, in the vast majority of cases, the immunological cross-reactions can be explained by side-chain sensitization [33]. 

### Various antibiotics 

Fusidic acid, a topical AB used to treat skin infections caused by Gram-positive bacteria, mainly *Staphylococcus aureus*, appears to rarely trigger CS [17, 34] and is therefore widely considered an alternative to topical aminoglycoside antibiotics. There is an increased risk of sensitization when fusidic acid is used to treat chronic leg ulcers, stasis dermatitis, and otitis externa [34]. 

Mupirocin is produced by *Pseudomonas fluorescens*, inhibits bacterial protein synthesis, and is effective against aerobic Gram-positive bacteria. Topical applications include treatment of skin infections and also elimination of staphylococci in the nasal vestibule. The occurrence of allergic CD is apparently very rare [35]. To date, only a few cases have been published, the first being in 1995 for a patient who had applied mupirocin topically for the treatment of chronic leg ulcers [35, 36]. In cases of CS to neomycin and/or bacitracin, mupirocin can be used as a safe alternative because it is the only representative of this pharmacologic class of agents and has a unique structure among topical ABs. Immunological cross-reactions have not been observed [16].

Metronidazole is a synthetic nitroimidazole derivative for the treatment of infections with anaerobic bacteria and protozoa. In addition, the substance has direct anti-inflammatory and immunosuppressive properties, which is why it is also used in the topical treatment of inflammatory dermatoses such as rosacea and perioral dermatitis. Topically, metronidazole is also used in the treatment of bacterial vaginosis, trichomoniasis, and occasionally in wound healing disorders of the skin. After intra-vaginal application as an ovule, drug reactions have been observed under the clinical picture of fixed-drug reaction [37] and SDRIFE (symmetrical drug-related intertriginous and flexural exanthema) [38]. Allergic CD on the face has been described in association with the use of metronidazole-containing topical preparations for the treatment of facial dermatoses such as rosacea and acneiform skin symptoms [39]. Cross-reactions to imidazole antifungals have been discussed. However, reliable data on this do not appear to exist to date [1, 40]. 

Chloramphenicol inhibits bacterial protein synthesis. Overall, topical use in Europe has decreased significantly in recent decades, and the sensitization potential is low. Allergic CD used to be induced via repeated application of chloramphenicol-containing eye drops [41]. 

Nitrofurazone (Nitrofural) is a broad-spectrum antibiotic from the nitrofuran group. Topical application used to be for the treatment of skin infections, ulcers, and burns. It is still marketed in Germany under the name “Furacin-Sol 0,2% Salbe”, approved for the treatment of superficial skin and wound infections. Due to a high incidence of allergic reactions, its use has been increasingly abandoned in Western countries [42]. Nitrofurazone was formerly used in veterinary medicine as an animal feed additive. Therefore, occupational exposure was generally present [42]. In the meantime, nitrofurans may no longer be used in food-producing animals in the EU, so that the substance is no longer important as an animal feed additive [43]. 

Oxytetracycline inhibits bacterial protein synthesis and is effective against many aerobic and anaerobic Gram-negative and Gram-positive bacteria, including *Rickettsia, Chlamydia, Mycoplasma*, and *Spirochetes*. Local application is also used for the treatment of acute and chronic bacterial infections of the anterior segment of the eye and superficial skin infections. CS with immunologic cross-reactions to other tetracyclines are generally possible [1]. Recent data on the current prevalence of CS to oxytetracycline are not available. 

Clioquinol is a halogenated hydroxyquinoline AB. It inhibits the growth of Gram-positive cocci (staphylococci, enterococci), various fungal pathogens (microsporon), Trichophyton, Candida albicans) and is also amebicidal. It was formerly used topically in the treatment of eczema and fungal infections. Sensitization has been observed in patients with chronic leg ulcers [1]. Clioquinol is a rare sensitizer and seems to be used much less frequently in recent years, not least because of the substance’s yellow intrinsic coloration 44]. Thus, based on data from the European Surveillance System on Contact Allergies (ESSCA), it was proposed in 2018 to remove clioquinol from the European Baseline Series [44]. Immunologic cross-reactions to other topical and systemic halogenated hydroxyquinolines, such as iodochlorhydroxyquin, iodoquinol, broxyquinoline, chlorquinaldol, and chlorhydroxyquinolines have been described [45]. In clioquinol-sensitized patients, positive epicutaneous patch test reactions to various antimalarials, such as quinine, chloroquine, and amodiaquine, have been observed [46]. 

Ozenoxacin is a bactericidal AB from the quinolone group, approved in 2017 in the USA and in 2019 as a 1% cream in Europe for the short-term treatment of non-bullous impetigo from 6 months of age. An increased risk of relevant CS has apparently not been observed so far [47]. 

Retapamulin is a semi-synthetic derivative of pleuromutilin with activity against staphylococci and streptococci. It is approved in Europe as a 1% topical preparation for the treatment of impetigo and minor infected wounds [3]. CA to retapamulin is apparently very rare and has been described only in isolated cases [48]. 

## Diagnosis and management of allergy to topical antibiotics 

**Figure 1:**
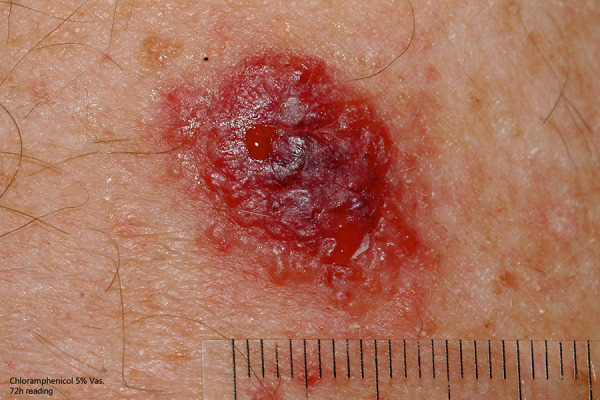
Epicutaneous patch test chloramphenicol 5% Vas.: +++ test reaction after 72 hours.

After CA has healed, epicutaneous patch testing is the most important tool for further allergological clarification (Figure 1). Current test concentrations of commercially available antibiotics according to recommendations of the German Contact Dermatitis Research Group (DKG) can be found in Table 2. In many cases, it is recommended to test not only the active ingredients but also the individual components such as preservatives, additives, and vehicles of the substances used [5]. Since a large proportion of the ABs in question are not available as commercial test preparations, it may be advisable to carry out testing with the patient’s own substances after informing the patient accordingly. The regulatory requirements of the German Medicinal Products Act (The Drug Law) (Arzneimittelgesetz, AMG) must be observed [49]. Since delayed patch test reactions often occur, readings after 1 week and/or later are indispensable in order to not overlook late reactions. 

After diagnosis, the patient must be informed about the substances to be avoided. This also includes information about possible immunological cross-reactions. 

## Conclusion 

Repeated or long-term use of topical antibiotics and an existing skin barrier defect are risk factors for the development of CA. In many cases, topical antibiotic treatment is not necessary with the use of modern antiseptics. Therefore, the indication must always be critically questioned not only because of the sensitization potential but also because of the unfavorable and potentially consequential resistance problem. 

## Funding 

None. 

## Conflict of interest 

J. Wohlrab has received grants for scientific projects, clinical studies, lectures and/or consulting from the following relevant companies in the last 5 years: Allergika, Almirall, Aristo, BayPharma, Dermapharm, Galderma, GSK, Helm, Hexal, Infectopharm, Jenapharm, Klinge, Leo, Medac, Medice, Mibe, MSD, Mylan, Novartis, Pierre Fabre, Pfizer, Skinomics, Wolff. 

B. Kreft has received grants for scientific projects, clinical studies, lectures or consulting in the last 5 years outside the submitted work from Jenapharm, Novartis, Biogen. 

**Table 1.** Antibiotics as triggers of contact allergies and their special features.

Antibiotics as triggers of contact allergies		Special features
Aminoglycoside antibiotics	Gentamicin Neomycin	In some countries, in combination preparations with steroids, antifungals, and/or bacitracin
Polypeptide antibiotics	Bacitracin	Occasional co-sensitization with neomycin by combined use
		“Late” test reactions in epicutaneous patch testing
		Immediate-type allergic reactions have also been described
	Polymyxin B	In some countries in combination preparations with bacitracin and neomycin.
		“Late” test reactions in epicutaneous patch testing
Lincosamide antibiotics	Clindamycin	Rare as a cause of allergic contact dermatitis
		Occasional atypical pictures of contact allergy (EEM-like, rosacea-like rash).
Macrolide antibiotics	Erythromycin	Very rare as a cause of allergic contact dermatitis
β-lactam antibiotics	Penicillins Cefalosporins	Contact allergy due to occupational exposure, e.g. during the preparation of infusions to be administered systemically
		Immediate-type allergic reactions possible
Various	Fusidic acid	Increased risk of contact allergy in patients with chronic leg ulcers, stasis dermatitis, otitis externa
	Mupirocin	Rarely a cause of allergic contact dermatitis
		Safe alternative in the case of contact sensitization to neomycin and bacitracin.
		No immunological cross reactions
	Metronidazole	Occasional atypical clinical pictures of type IV allergy (fixed AME, SDRIFE).
	Chloramphenicol	In the past, allergic contact dermatitis often via application in eye drops
		Topical use in Europe rare nowadays
	Nitrofurazone	In the past, occupational exposure via animal feed additive
		Still marketed in Germany (furacin-sol 0.2% ointment)
	Oxytetracycline	No data on current prevalence of contact sensitization
		Immunological cross-reactions to other tetracyclines possible
	Clioquinol	Contact sensitization is rare
		Immunological cross-reactions to other halogenated hydroxyquinolines have been described
	Ozenoxacin	Approved 2019 as a topical antibiotic in Europe
		So far, no increased risk of contact sensitization
	Retapamulin	Contact allergies described so far only in isolated cases

**Table 2.** Test concentrations of various commercially available antibiotics according to recommendations of the German Contact Allergy Group (DKG) – as of January 2022 (https://dkg.ivdk.org/testreihen.html#a005).

Active ingredient	Test concentration	Vehicle (Vas.: Vaseline)
Bacitracin	20%	Vas.
Gentamicin sulfate	20%	Vas.
Oxytetracycline	3%	Vas.
Framycetin sulfate	10%	Vas.
Fusidic acid (Na. salt)	2%	Vas.
Neomycin sulfate	20%	Vas.
Polymyxin B sulfate	3%	Vas.
Chloramphenicol	5%	Vas.
Kanamycin sulfate	10%	Vas.
Clioquinol (Iodochlorhydroxyquin)	5%	Vas.
